# Pre-treatment prognostic nutritional index may serve as a potential biomarker in urinary cancers: a systematic review and meta-analysis

**DOI:** 10.1186/s12935-018-0708-7

**Published:** 2018-12-17

**Authors:** Feng Qi, Xiang Zhou, Yi Wang, Yamin Wang, Yichun Wang, Qijie Zhang, Rong Cong, Jie Yang, Ninghong Song

**Affiliations:** Department of Urology, The First Affiliated Hospital of Nanjing Medical University, No. 300 Guangzhou Road, Nanjing, 210009 China

**Keywords:** Prognostic nutritional index, PNI, Urinary cancer, Meta-analysis

## Abstract

**Background:**

To investigate the potential prognostic role of pre-treatment prognostic nutritional index (PNI) in urinary cancers.

**Methods:**

Relevant articles were searched comprehensively from PubMed, Embase and Web of Science, up to November 2018. The pooled hazard ratios (HRs) with 95% confidence intervals (CIs) were extracted to evaluate their associations.

**Result:**

A total of 12 related articles including 6561 patients were ultimately enrolled. Our results indicated that a relatively lower level of pre-treatment PNI was associated with decreased OS, CSS/DSS and DFS/RFS/PFS (pooled HR = 1.68, 95% CI 1.45–1.95; pooled HR = 1.57, 95% CI 1.33–1.86; pooled HR = 1.75, 95% CI 1.53–1.99, respectively). Subsequent stratified analysis by cancer type for OS showed that PNI could also be a predictor no matter in renal cell cancer (RCC) or bladder cancer (BC) (pooled HR = 1.65, 95% CI 1.37–1.97 and pooled HR = 1.67, 95% CI 1.20–2.33). Similar results could be found in DFS/RFS/PFS (RCC: HR = 1.81, 95% CI 1.54–2.13 and BC: HR = 1.68, 95% CI 1.32–2.12) and in CSS/DSS (RCC: HR = 1.50, 95% CI 1.23–1.82 and upper tract urothelial carcinoma: HR = 1.61, 95% CI 1.13–2.28). As for the treatment subgroup, a relatively lower level of PNI could also be a positive predictor for OS (surgery: HR = 1.64, 95% CI 1.40–1.93; target therapy: HR = 1.88, 95% CI 1.34–2.63) and DFS/RFS/PFS (surgery: HR = 1.69, 95% CI 1.47–1.95; target therapy: HR = 2.14, 95% CI 1.50–3.05).

**Conclusion:**

The outcomes of us shed light on that elevated pre-treatment PNI was positively associated with OS, CSS/DSS and DFS/RFS/PFS, indicating that it could be an independent prognostic factor in urinary cancers.

## Background

Urinary cancers, as a term mainly consisting of prostate cancer (PC), bladder cancer (BC), renal cell cancer (RCC), are much more common in men than in women and the incidence of these tumors ranks the second, fifth and seventh most commonly diagnosed cancer in the United States, 2017 [1]. Generally, the mainstay of therapy for localized urological tumors is surgical resection, while target therapy is mainly for metastatic cases. Due to the appearance of sipuleucel-T based immunotherapy and the development of molecular target drugs [2, 3], survival of urinary cancer has been greatly improved [4]. However, the prognosis of these tumors is still not satisfying. As for RCC, postoperative recurrence occurs in one-third of patients [5]. Meanwhile, in terms of BC, its 5-year survival remains 77.9%, even only 5.4% for distant diseases [6]. Therefore, exploring the prognostic factors for survival, death or recurrence may be of great value to better understand these and help physicians to develop the optimal treatment strategies for patients.

Prognostic nutritional index (PNI), as a predictor of cancer prognosis, was firstly introduced by Onodera et al. [7] to investigate the potential prognostic role in gastrointestinal malignancy in 1984. Moreover, it had also been validated to be an independent prognostic factor in many other types of tumors, such as hepatocellular carcinoma [8], pancreatic cancer [9], and pleural mesothelioma [10]. Recently, PNI as a prognostic factor in the case of urinary cancer, had gradually gained a lot of interest and accumulating researches considered it to be an independent prognostic factor in urinary tumors, associated with overall survival (OS), progression-free survival (PFS) or cancer specific survival (CSS). However, their results remained inconsistent. Hence, this meta-analysis was conducted systematically to shed light on the relationship between PNI and urinary cancer. Due to the absence of level I evidence guiding the application of PNI in urinary cancers, our results were also anticipated to provide some references for clinical work.

## Materials and methods

### Search strategy

To investigate the potential role of PNI in urinary cancers, relevant articles were searched comprehensively from online databases PubMed, Embase and Web of Science, up to November 2018. The search strategy was consisted of the following keywords in combination with Medical Subject Headings (MeSH) terms and text words: (“prognostic nutritional index” or “PNI”) and (“urological tumors” or “prostate cancer” or “renal cell cancer” or “bladder cancer” or “urothelial cancer” or “upper tract urothelial carcinoma”) and (“survival” or “recurrence” or “prognosis” or “progress”). This meta-analysis was performed according to the preferred reporting items for systematic reviews and meta-analyses (PRISMA) statement [11] and no language restriction was applied in the selection process.

### Inclusion/exclusion criteria

Articles eventually enrolled in this meta-analysis should meet the following criteria: (1) cohort studies or case–control studies; (2) patients were diagnosed with urinary cancers histopathologically; (3) the association of pre-treatment PNI with specific endpoint (e.g. OS, recurrence-free survival (RFS), PFS, disease-free survival (DFS), disease-specific survival (DSS) or CSS; (4) available data by means of hazard ratios (HRs) with 95% confidence interval (CIs) should be provided. Exclusion criteria were as follows: (1) lack of accurate data; (2) letter, review and case report; (3) simple description without comparison. Additionally, only the largest sample size study was included if the same series of research were used in various articles.

### Data extraction and quality assessment

The whole selection process was performed independently by two blind investigators (F.Q and X.Z). Disagreements were addressed by consultation with a third reviewer (Y.W). Following data were extracted from articles based on standard form: first author’s name, year of publication, country, cancer type, study design, treatment methods, sample size (number of total patients), PNI cut-off values, endpoints, HRs with 95% CIs and follow-up. Data were extracted from Kaplan–Meier curves to extrapolate HRs with 95% CIs using previously described methods, if it could not be directly obtained from each article [12, 13]. Methodologic quality of each included articles was assessed by the Newcastle–Ottawa Scale (NOS) (http://www.ohri.ca/programs/clinical_epidemiology/oxford.htm), which was one of the most useful scale to evaluate the quality of non-randomized studies [14]. Total quality scores were ranged from 0 to 9 and if the final score > 6, we regarded it to be of high quality. Detailed rankings for each study were shown in Table 1.

**Table 1:** Newcastle–Ottawa quality assessments scale

Studies	Year	Quality indicators from Newcastle–Ottawa Scale								Scores
		1	2	3	4	5	6	7	8	
Cai [18]	2017	★	★	–	–	★★	–	★	★	6
Peng [25]	2017	–	★	★	–	★★	★	★	★	7
Miyake [24]	2017	★	★	–	★	★★	–	★	–	6
Cui [23]	2017	–	★	–	★	★★	★	–	★	6
Huang [26]	2017	★	–	★	★	★★	–	★	★	7
Fan [28]	2017	★	★	–	★	★★	–	★	★	7
Broggi [17]	2016	★	–	★	★	★★	–	★	–	6
Kwon [21]	2017	★	★	–	★	★★	★	★	–	7
Peng [22]	2017	★	–	–	★	★★	–	★	★	6
Jeon [20]	2016	★	★	–	★	★★	★	–	★	7
Kim [27]	2015	–	★	★	–	★★	★	★	★	7
Hofbauer [19]	2015	★	–	★	★	★★	–	★	★	7

1. Representativeness of the exposed cohort; 2. Selection of the non-exposed cohort; 3. Ascertainment of exposure; 4. Outcome of interest not present at start of study; 5. Control for important factor or additional factor; 6. Assessment of outcome; 7. Follow-up long enough for outcomes to occur; 8. Adequacy of follow up of cohorts

### Statistical analysis

To investigate the potential role of PNI in urinary cancers, this meta-analysis was conducted based on available data and the pooled HRs with 95% CIs were utilized to evaluate their efficacy. Cochran’s Q test and Higgins I^2^ statistic were used to evaluate the heterogeneity. If significant heterogeneity (*P* < 0.10 or *I*^2^ > 50%) existed, the random effect model (a DerSimonian-Laird method) would be applied. Otherwise, the fixed effect model (a Mantel–Haenszel method) was adopted [15]. The stability and reliability of the results was determined by sensitive analysis, which was an effective measure to recount the pooled ORs via consecutively excluding each study once a time. Furthermore, publication bias was assessed by Begg’s funnel plot and Egger’s linear regression test, and *P* < 0.05 was considered to be statistically significant [16]. In addition, all statistical data were conducted by Stata software (version 12.0; StataCorp LP, College Station, TX) and Microsoft Excel (V.2007, Microsoft Corporation, Redmond, WA, USA).

## Results

### Study characteristics

**Fig. 1:**
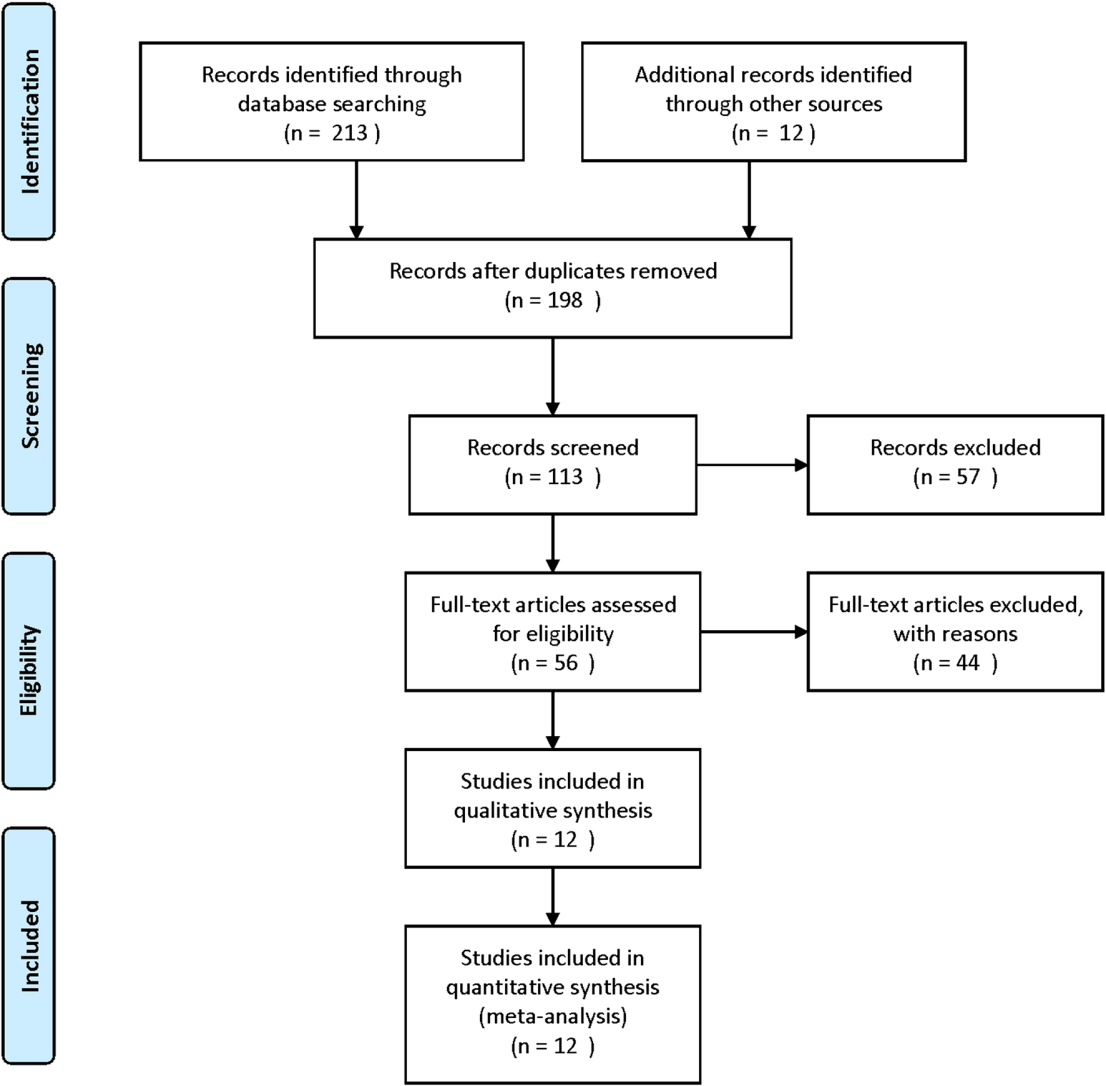
Flow diagram of literature search and selection process

A total of 12 [17–28] articles including 6561 patients were ultimately involved after systematic selection. Flow diagram of literature search and selection process was summarized in Fig. 1. The NOS scores of enrolled articles were all above 6 (Table 1) and the basic characteristics of eligible studies were present in Table 2. All of the studies were retrospective. In terms of tumor type, 6 articles focused on RCC, 3 articles focused on BC, 2 articles focused on upper tract urothelial carcinoma (UTUC) and only 1 article focused on PC. In the case of treatment type, 9 articles were on surgery and 3 articles were on target therapy. Of all the 12 articles,9 articles investigated the prognostic role of PNI for OS, 3 for CSS, 3 for DFS, 5 for PFS, 2 for RFS and 2 for DSS. In addition, the cut-off value of PNI applied in each study was varied from each other, ranged from 44.7 to 52.57.

**Table 2:** Main characteristics of individual studies included in the meta-analysis

Study	Year	Country	Cancer type	Study design	Treatment	Number of patients	Cut-off values	HR (95% CI)	Months of follow-up
Overall survival (OS)									
Cai [18]	2017	China	RCC	Retrospective	Target therapy	178	51.62	1.658 (1.04–2.641)	22 months median
Peng [25]	2017	China	BC	Retrospective	RC	516	46.025/47.2	1.668 (1.147–2.425)	37 months median
Miyake [24]	2017	Japan	BC	Retrospective	RC	117	50	1.70 (0.80–3.30)	22 months median
Huang [26]	2017	China	UTUC	Retrospective	RNU	425	46.78	1.74 (1.20–2.53)	38.5 months median
Fan [28]	2017	China	PC	Retrospective	Target therapy	112	50.5	3.80 (1.00–13.9)	20.2 months median
Broggi [17]	2016	American	RCC	Retrospective	Nephrectomy	341	44.7	1.73 (1.09–2.76)	NR
Kwon [21]	2017	Korea	RCC	Retrospective	Target therapy	125	41	1.96 (1.16–3.33)	45.3 months median
Peng [22]	2017	China	RCC	Retrospective	Nephrectomy	1360	47.625/47.775	1.645 (1.153–2.348)	67 months median
Jeon [20]	2016	Korea	RCC	Retrospective	PN, RN	1437	51	1.50 (1.09–2.07)	68.6 months mean
Progression/disease/recurrence-free survival (PFS/DFS/RFS)									
Cai [18]	2017	China	RCC	Retrospective	Target therapy	178	51.62	1.842 (1.226–2.766)	22 months median
Peng [25]	2017	China	BC	Retrospective	RC	516	46.025/47.2	1.68 (1.092–2.005)	37 months median
Cui [23]	2017	China	BC	Retrospective	TURBT	329	52.57	1.672 (1.149–2.439)	43.9 ± 27.1 (mean ± SD)
Fan [28]	2017	China	PC	Retrospective	Target therapy	112	50.5	3.50 (1.10–11.0)	20.2 months median
Broggi [17]	2016	American	RCC	Retrospective	Nephrectomy	341	44.7	2.26 (1.42–3.73)	NR
Kwon [21]	2017	Korea	RCC	Retrospective	Target therapy	125	41	3.33 (1.35–8.33)	45.3 months median
Peng [22]	2017	China	RCC	Retrospective	Nephrectomy	1360	47.625/47.775	1.705 (1.266–2.296)	67 months median
Kim [27]	2015	Korea	UTUC	Retrospective	NU	277	45	1.183 (0.656–2.132)	57.2 months median
Jeon [20]	2016	Korea	RCC	Retrospective	PN, RN	1437	51	1.47 (1.03–2.11)	68.6 months mean
Hofbauer [19]	2015	Austria	RCC	Retrospective	PN, RN	1344	48	1.96 (1.32–2.86)	40 months median
Cancer/disease-specific survival (CSS/DSS)									
Miyake [24]	2017	Japan	BC	Retrospective	RC	117	50	3.30 (1.40–7.40)	22 months median
Huang [26]	2017	China	UTUC	Retrospective	RNU	425	46.78	1.98 (1.31–2.99)	38.5 months median
Jeon [20]	2016	Korea	RCC	Retrospective	PN, RN	1437	51	1.51 (1.05–2.19)	68.6 months mean
Kim [27]	2015	Korea	UTUC	Retrospective	NU	277	45	0.947 (0.491–1.826)	57.2 months median
Hofbauer [19]	2015	Austria	RCC	Retrospective	PN, RN	1344	48	1.49 (1.19–1.89)	40 months median

*HR* hazard ratio, *CI* confidence interval, *RCC* renal cell cancer, *BC* bladder cancer, *UTUC* upper tract urothelial carcinoma, *PC* prostate cancer, *RC* radical cystectomy, *PN* partial nephrectomy, *RN* radical nephrectomy, *TURBT* transurethral resection of bladder tumor, *NU* nephrouretectomy, *RNU* radical nephrouretectomy, *NR* not reported

### OS associated with PNI in urinary cancer

**Fig. 2:**
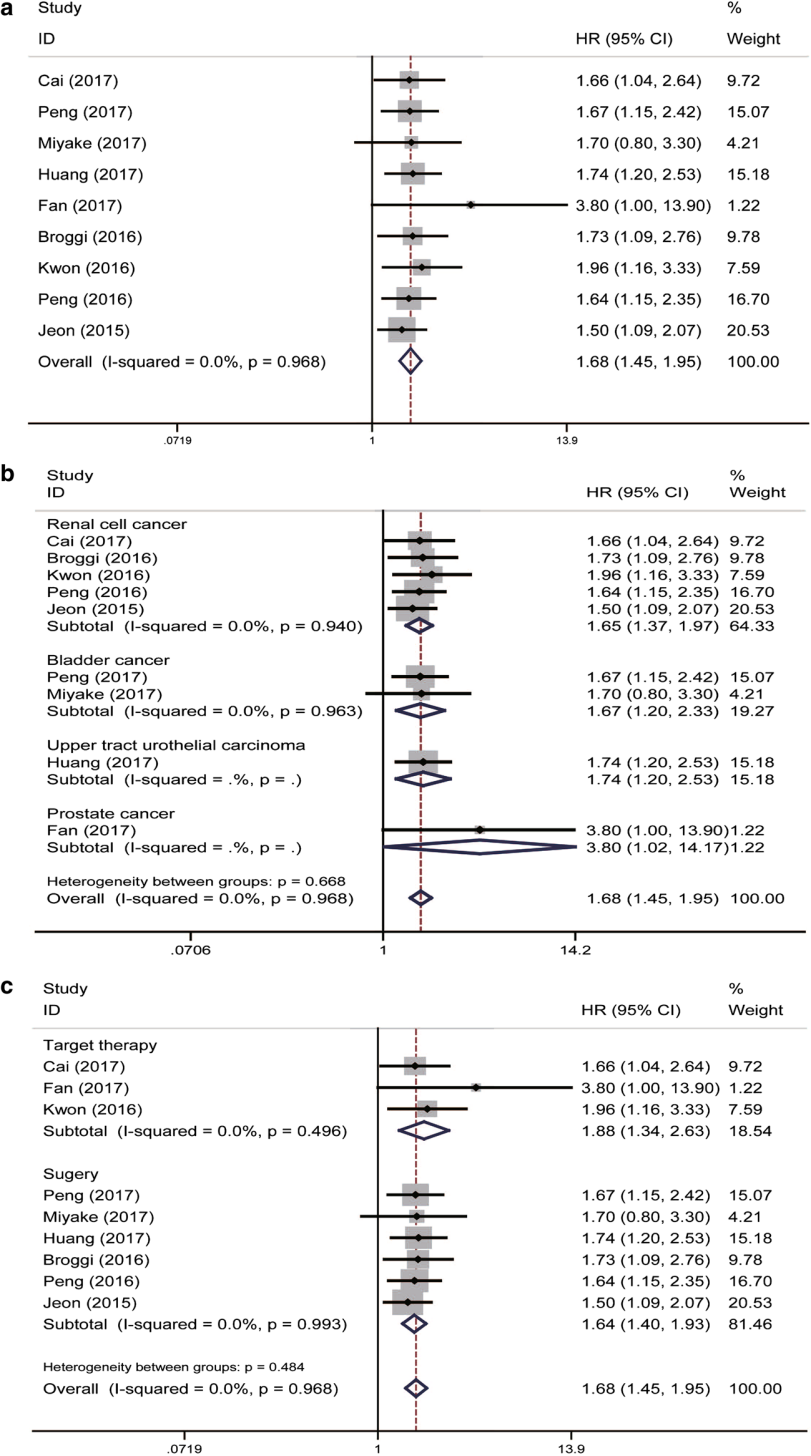
Forrest plots of OS associated with PNI in urinary cancers. **a** The overall group; **b** the subgroup analysis of cancer type; **c** the subgroup analysis of treatment type

A total of nine eligible studies revealed the prognostic role of pre-treatment PNI in urinary cancer on OS by fixed-effects model with no heterogeneity (*P* = 0.968, *I*^2^ = 0.0%). Our results indicated that a relatively lower level of pre-treatment PNI was associated with decreased OS (pooled HR = 1.68, 95% CI 1.45–1.95) (Fig. 2a). Subsequent stratified analysis by cancer type for OS indicated that PNI could also be a positively predictor in RCC, BC, UTUC and PC (pooled HR = 1.65, 95% CI 1.37–1.97, *P* = 0.940, *I*^2^ = 0.0%; pooled HR = 1.67, 95% CI 1.20–2.33, *P *= 0.963, *I*^2^ = 0.0%; pooled HR = 1.74, 95% CI 1.20–2.53; pooled HR = 3.80, 95% CI 1.02–14.17; respectively) (Fig. 2b). As for the treatment subgroup, a relatively lower level of PNI could be a positively predictor for OS (Surgery: HR = 1.64, 95% CI 1.40–1.93, *P* = 0.993, *I*^2^ = 0.0%; target therapy: HR = 1.88, 95% CI 1.34–2.63, *P* = 0.496, *I*^2^ = 0.0%; separately) (Fig. 2c).

### DFS/RFS/PFS associated with PNI in urinary cancer

**Fig. 3:**
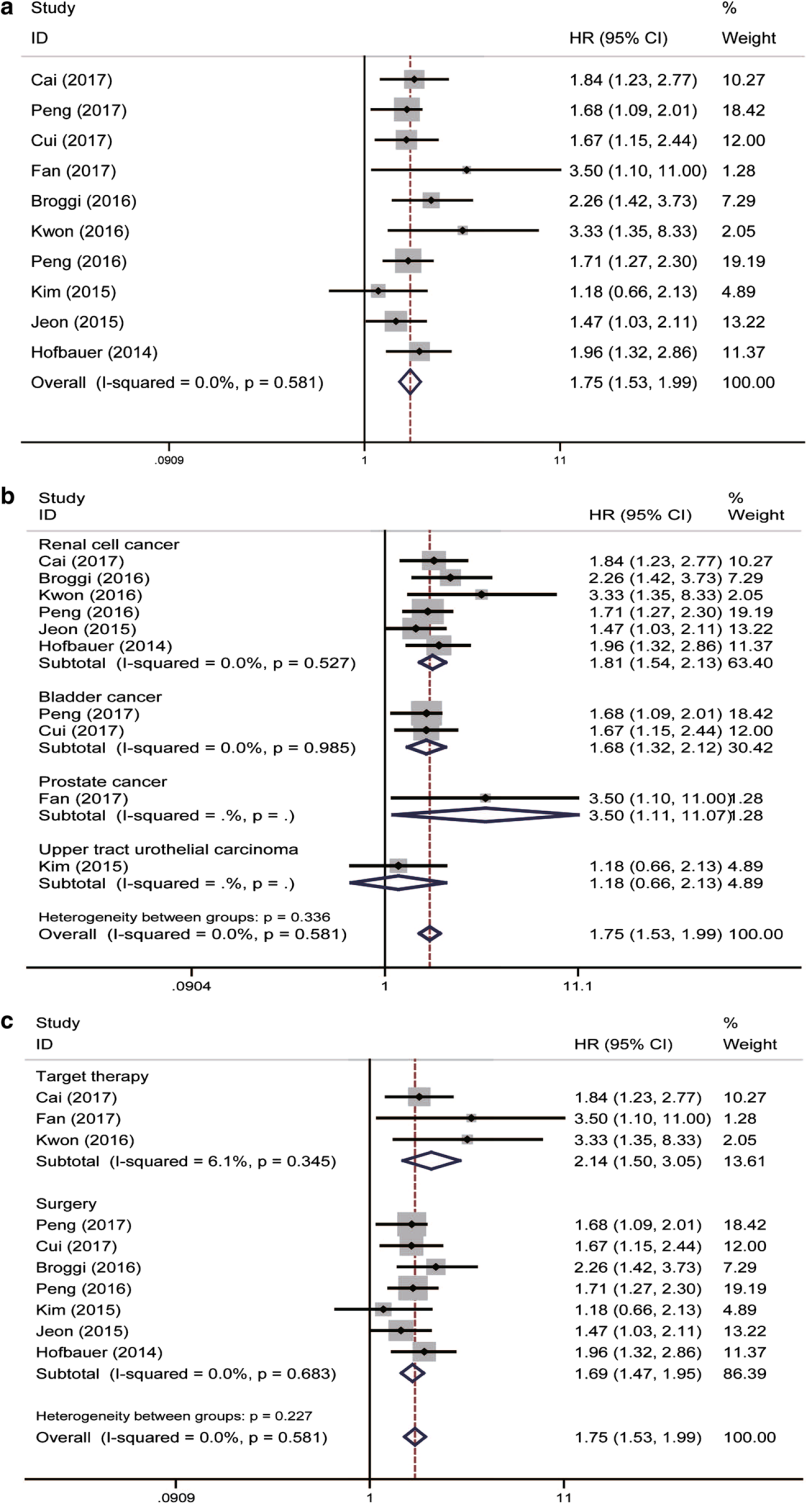
Forrest plots of DFS/RFS/PFS associated with PNI in urinary cancers. **a** The overall group; **b** the subgroup analysis of cancer type; **c** the subgroup analysis of treatment type

A total of ten eligible studies revealed the prognostic role of pre-treatment PNI in urinary cancers on DFS/RFS/PFS by fixed-effects model with no heterogeneity (*P* = 0.581, *I*^2^ = 0.0%). Our results indicated that a relatively lower level of pre-treatment PNI was associated with decreased DFS/RFS/PFS (pooled HR = 1.75, 95% CI 1.53–1.99) (Fig. 3a). Subsequent stratified analysis by cancer type for DFS/RFS/PFS showed that PNI could also be a positively predictor in RCC, BC and PC (pooled HR = 1.81, 95% CI 1.54–2.13, *P *= 0.527, *I*^2^ = 0.0%; pooled HR = 1.68, 95% CI 1.32–2.12, *P* = 0.985, *I*^2^ = 0.0%; pooled HR = 3.50, 95% CI 1.11–11.07; respectively) (Fig. 3b). In the case of the treatment subgroup, a relatively lower level of PNI could be a positively predictor for DFS/RFS/PFS (surgery: HR = 1.69, 95% CI 1.47–1.95, *P* = 0.683, *I*^2^ = 0.0%; target therapy: HR = 2.14, 95% CI 1.50–3.05, *P *= 0.345, *I*^2^ = 6.1%; separately) (Fig. 3c).

### CSS/DSS associated with PNI in urinary cancer

**Fig. 4:**
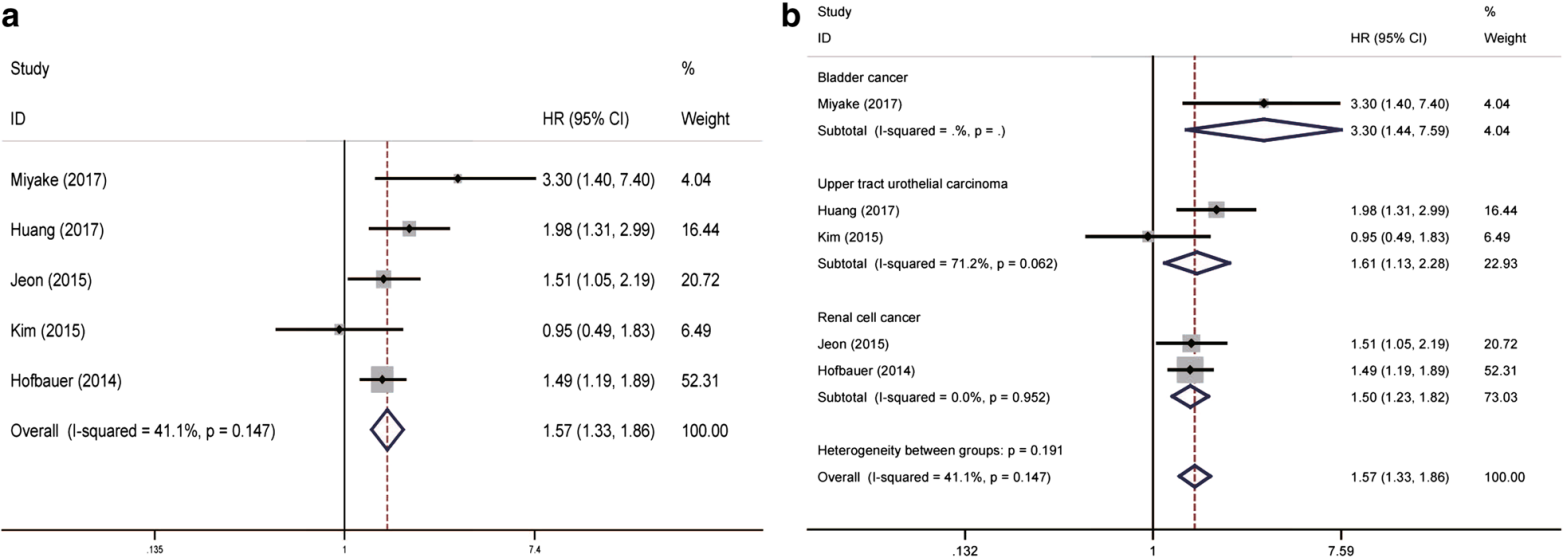
Forrest plots of CSS/DSS associated with PNI in urinary cancers. **a** The overall group; **b** the subgroup analysis of cancer type

A total of five eligible studies revealed the prognostic role of pre-treatment PNI in urinary cancer on CSS/DSS by fixed-effects model with moderate heterogeneity (*P* = 0.147, *I*^2^ = 41.1%). Our results indicated that a relatively lower level of pre-treatment PNI was associated with decreased CSS/DSS (pooled HR = 1.57, 95% CI 1.33–1.86) (Fig. 4a). Subsequent stratified analysis by cancer type for CSS/DSS showed that PNI could also be a positively predictor in RCC, UTUC and BC (pooled HR = 1.50, 95% CI 1.23–1.82, *P* = 0.952, *I*^2^ = 0.0%; pooled HR = 1.61, 95% CI 1.13–2.28, *P* = 0.062, *I*^2^ = 71.2%; pooled HR = 3.30, 95% CI 1.44–7.59; respectively) (Fig. 4b).

### Sensitivity analysis

**Fig. 5:**
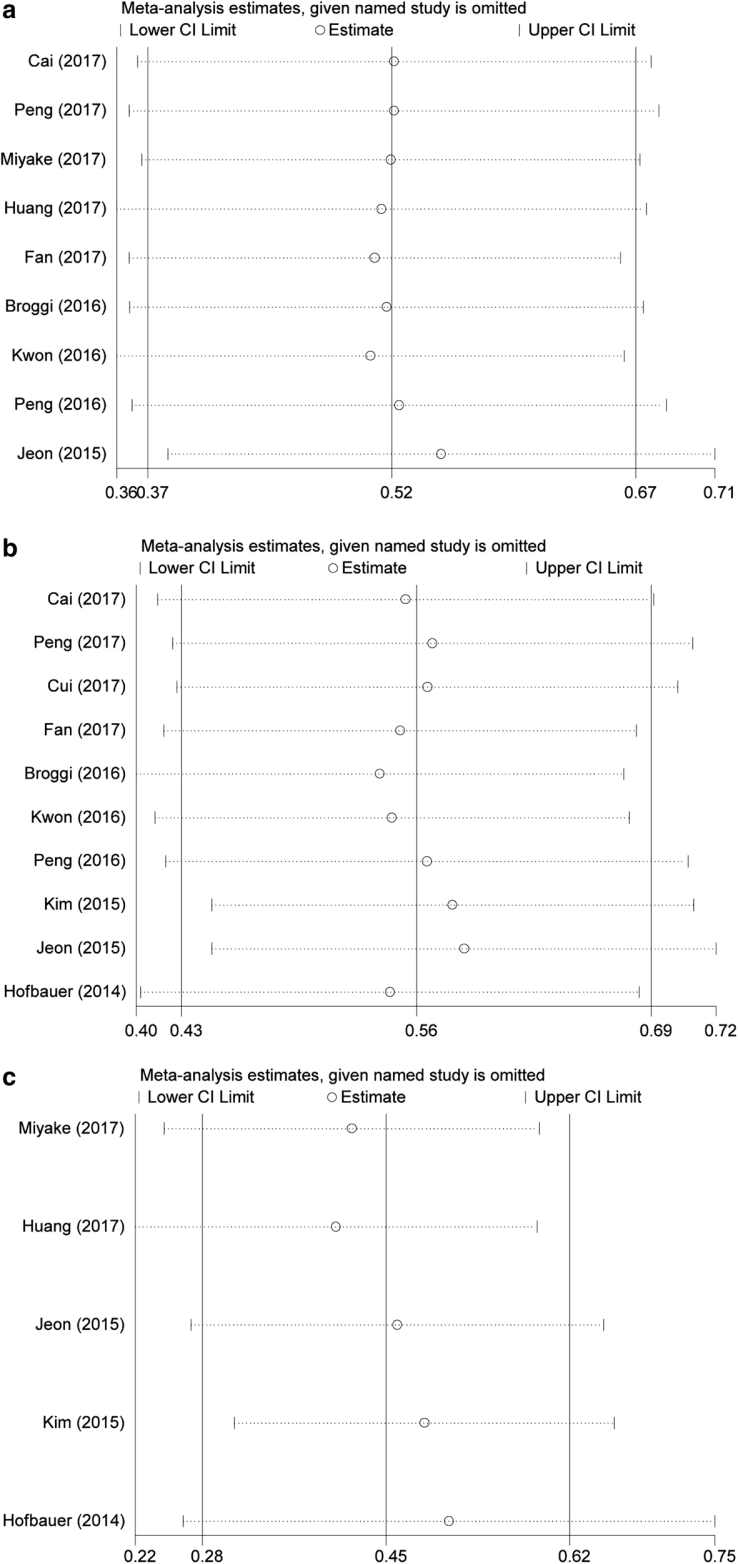
Sensitivity analysis of each included study. **a** OS for individual studies; **b** DFS/RFS/PFS for individual studies; **c** CSS/DSS for individual studies

Sensitivity analysis was assessed by calculating the remained part by omitting one single study once a time to reflect the impact of the individual to overall. The sensitivity analysis of the results for pre-treatment PNI in urinary cancers indicated that no single study significantly influenced the pooled OR and 95% CIs. Namely, our results were robust (Fig. 5).

### Publication bias

**Fig. 6:**
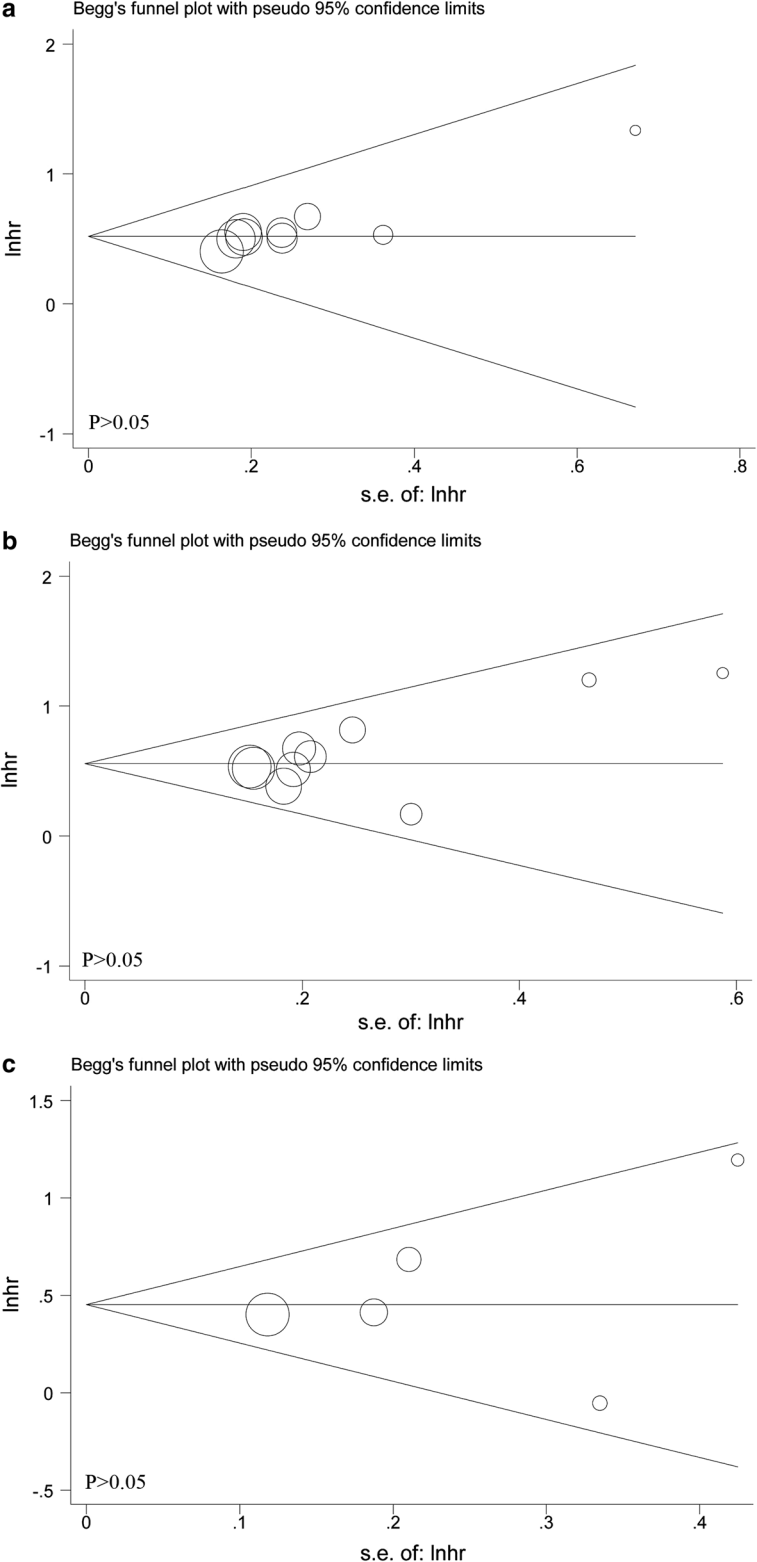
Begg’s funnel plots of the publication bias. **a** OS for individual studies; **b** DFS/RFS/PFS for individual studies; **c** CSS/DSS for individual studies

As displayed in Fig. 6, publication bias was accessed by the combined application of Begg’s and Egger’s test. In the pooled analysis of OS or DFS/RFS/PFS or CSS/DSS, the *P* values of them were all above 0.05, indicating no significant bias was identified. In other words, our results were reliable based on the available articles.

## Discussion

Urinary cancers had accounted for a relatively large proportion of all tumors and the newly estimated cases of PC, RCC and BC were 161,360, 63,990 and 79,030 respectively in USA, 2017 [1]. Metastases or postoperative recurrence were highly likely to occur in these tumors, for example, approximately 75% high-risk bladder cancer patients would recur, progress, or die within 10 years after their initial diagnosis [29]. Moreover, up to 20% of all RCC patients would lead to local or distant disease recurrence ultimately [30]. Once metastasized, the 5-year survival rate was less than 10% [31]. Obviously, it was utmostly important to identify the prognostic factors in urinary tumors. To our best knowledge, it was the first meta-analysis to estimate the prognostic role of pre-treatment PNI in urinary cancers.

Accumulating data had been widely investigated for a long time on the prediction of tumor survival and recurrence. The host inflammatory response had already been proved to be a predictor of survival independent of stage and grade in many solid tumors [32, 33]. Existing hypothesis claimed that this process was suitable for the tumor growth in their microenvironment, based on its provision of growth factors, proangiogenic factors or extracellular matrix enzymes [34]. On the other hand, the cancer stem cell pathway could also be activated by inflammatory cytokines, which could promote the development and invasion of the tumor [35]. In terms of these, the prognostic role of C-reactive protein in RCC had been confirmed [36]. Furthermore, the host nutritional status was considered to be closely related to tumor prognosis. In 2009, Karl et al. [37] made an evaluation in 897 urologic patients utilizing the Nutritional Risk Screening 2002 (NRS), claimed that 16% of patients were under the risk of malnutrition, which can contribute to malignant disease. Gregg et al. [38] found a simple model, measured by body mass index (BMI), serum albumin and preoperative weight loss, that which can predict 90-day mortality and poor OS at 3 years in BC patients. Additionally, a study conducted by Lambert et al. [39] demonstrated that the pre-treatment albumin levels had something to do with higher mortality. However, there is no unified and approved standard to reflect the nutritional status of preoperative patients.

Past researches had revealed that many factors could be investigated to predict the prognosis in urinary tumors. Pre-treatment neutrophil-to-lymphocyte (NLR) ratio and pre-treatment lymphocyte-monocyte ratio (LMR) had been proved to be an independent prognostic factor in various urinary cancers [40–42]. Generally, high pre-treatment NLR or LMR was closely associated with poor survival. Santoni et al. [43] thought pre-treatment NLR to be an independent prognostic factor for mRCC patients treated with second- or third-line everolimus. Yoshida et al. [44] found that pre-treatment lower level LMR could predict poorer OS and CSS by analyzing 302 patients underwent radical cystectomy. Meanwhile, the potential role of LMR may superior to NLR to some extent. Similarly, the potential role of Glasgow prognostic score (GPS) [45] and systemic immune-inflammation index (SII) [46] had also been explored.

In this meta-analysis, conclusion could be drawn that a relatively lower pre-treatment PNI was tightly associated with a poorer OS, DFS/PFS/RFS and CSS/DSS. Subgroup analysis by cancer type or treatment type showed the similar results. PNI, calculated by serum albumin levels and lymphocyte count and its accurate value equals to 10 * serum albumin concentration (g/dL) + 0.005 * lymphocyte counts (number/mm^2^), was first applied by Onodera et al. [7] to assess the nutritional and immunological status of gastrointestinal surgical patients. Serum albumin, known as an indicator of host inflammatory and nutritional status, had been verified its prognostic role in various types of cancers [47, 48]. In addition, the host immune response activated by lymphocytes can help clearance of the tumor cells or prevent them from developing [34], the lower level of lymphocytes may represent a poorer survival or a higher mortality [49]. Therefore, it was easy to explain that why the PNI level, determined by serum albumin and lymphocytes, played an important role in prognosis of urinary tumors. In our study, a lower level of PNI may indicate a poorer survival and higher possibility of recurrence in urinary tumors regardless of its tumor type and treatment.

During our selection process, only one article focused on the relationship between PNI and prostate cancer [28]. In that research, Fan and his team assessed the prognostic role of PNI in prostate cancer treated with abiraterone (AA), a high baseline PNI level was tightly related to the initial response to AA treatment in metastatic castration-resistant prostate cancer patients (mCRPC), and the lower PNI level may predict poorer OS, radiographic PFS (rPFS), PSA-PFS. Furthermore, add PNI into the prediction model could increase the accuracy of a multivariate model for OS.

The strength of our study was mainly its strict inclusion criteria for eligible studies and the entire heterogeneity was relatively low. In addition, it was the first time for us to shed light on the prognostic role of pre-treatment PNI in urinary cancers. Nonetheless, several potential limitations should be paid attention to before fully understanding this study. Firstly, uncontrollable bias may exist because all of the included articles were retrospective studies rather than randomized controlled trials. Secondly, related articles were too few to obtain a reliable result in some specific endpoints (e.g. only five articles included in the analysis for CSS/DSS). Due to the relatively small sample size, unavoidable bias might also exist. Thirdly, most of the included articles were from Asia which may make the subgroup analysis hard to be performed. Last but not least, upcoming prospective RCTs were required to provide more available data and subsequent researches should resolve the aforementioned difficulties before pretreatment PNI was widely used in clinical practice.

## Conclusion

In summary, the outcomes of this meta-analysis shed light on that a higher level of pre-treatment PNI was positively associated with OS, CSS/DSS and DFS/RFS/PFS, indicating that it could be an independent prognostic factor in urinary tumors. Due to the limited researches, it restricted our in-depth investigation of the role of PNI. Hence, larger-samples with higher-quality randomized controlled trials were required to verify our findings.
